# Repeated intranasal TLR7 stimulation reduces allergen responsiveness in allergic rhinitis

**DOI:** 10.1186/1465-9921-13-53

**Published:** 2012-06-22

**Authors:** Lennart Greiff, Anders Cervin, Cecilia Ahlström-Emanuelsson, Gun Almqvist, Morgan Andersson, Jan Dolata, Leif Eriksson, Edward Högestätt, Anders Källén, Per Norlén, Inga-Lisa Sjölin, Henrik Widegren

**Affiliations:** 1 Department of ORL, Head & Neck Surgery, Skane University Hospital, Lund, Sweden; 2 Department of ORL, Helsingborg Hospital, Helsingborg, Sweden; 3 Research & Development, AstraZeneca, Lund, Sweden; 4 Department of Clinical Pharmacology, Skane University Hospital, Lund, Sweden

**Keywords:** Allergy, Immunity, Seasonal, Toll-like receptor 7, Treatment

## Abstract

**Background:**

Interactions between Th1 and Th2 immune responses are of importance to the onset and development of allergic disorders. A Toll-like receptor 7 agonist such as AZD8848 may have potential as a treatment for allergic airway disease by skewing the immune system away from a Th2 profile.

**Objective:**

To evaluate the efficacy and safety of intranasal AZD8848.

**Methods:**

In a placebo-controlled *single ascending dose study*, AZD8848 (0.3-600 μg) was given intranasally to 48 healthy subjects and 12 patients with allergic rhinitis (NCT00688779). In a placebo-controlled *repeat challenge/treatment study*, AZD8848 (30 and 60 μg) was given once weekly for five weeks to 74 patients with allergic rhinitis out of season: starting 24 hours after the final dose, daily allergen challenges were given for seven days (NCT00770003). Safety, tolerability, pharmacokinetics, and biomarkers were monitored. During the allergen challenge series, nasal symptoms and lavage fluid levels of tryptase and α_2_-macroglobulin, reflecting mast cell activity and plasma exudation, were monitored.

**Results:**

AZD8848 produced reversible blood lymphocyte reductions and dose-dependent flu-like symptoms: 30–100 μg produced consistent yet tolerable effects. Plasma interleukin-1 receptor antagonist was elevated after administration of AZD8848, reflecting interferon production secondary to TLR7 stimulation. At repeat challenge/treatment, AZD8848 reduced nasal symptoms recorded ten minutes after allergen challenge up to eight days after the final dose. Tryptase and α_2_-macroglobulin were also reduced by AZD8848.

**Conclusions:**

Repeated intranasal stimulation of Toll-like receptor 7 by AZD8848 was safe and produced a sustained reduction in the responsiveness to allergen in allergic rhinitis.

**Trial registration:**

NCT00688779 and NCT00770003 as indicated above.

## Background

As suggested by the hygiene hypothesis, infections are of importance to the maturation of the immune system [1]. Th1-mediated immunity may be defective in a modern clean environment resulting in facilitation of Th2 responses associated with allergic disorders [2,3]. Conversely, up-regulated Th1 responses, e.g. as a consequence of infections, can be associated with reduced Th2 activity and reduced responsiveness to allergen [4]. Controlled infection-like stimulation of the immune system may in this context be beneficial, and may be achieved by the use of Toll-like receptor (TLR) agonists.

TLRs are receptors of the innate immune system that recognise conserved microbial components known as pathogen-associated molecular patterns (PAMPs) [5]. PAMPs include the bacterial product LPS, viral single-stranded RNA, and bacterial/viral CpG DNA, acting as TLR4, TLR7, and TLR9 ligands, respectively [6]. Activation of TLRs stimulates the innate immune system, potentially leading to down regulation of Th2 adaptive responses to allergen [6]. The possibility of skewing the immune system away from a Th2 response, as has been attempted previously by other measures [7-10], is the basis for the development of TLR agonists as treatments for allergic rhinitis and asthma.

In a murine model of allergic asthma, a TLR7 ligand (S28463) administered systemically exerted anti-allergic effects resulting in attenuated airway eosinophilia, normalized airway responsiveness, and prevention of airway remodelling [11,12]. In a similar model, Sel *et al*. [13] demonstrated that systemic intervention with poly(I:C) and R-848, viral ligands recognized by TLR3 and TLR7 respectively, prevented production of allergen specific IgE and IgG1 during sensitisation and subsequently alleviated experimental asthma. Moreover, administration of poly(I:C) and R-848 in established allergy markedly reduced the responsiveness to allergen [13]. Similarly, the TLR9 ligand 1018 ISS was shown to inhibit Th2-mediated airway inflammation and hyperresponsiveness in animals [14-18].

AZD8848 is a selective TLR7 agonist optimised for topical airway treatment through rapid metabolism by plasma esterases, thereby reducing systemic exposure [19]. Observations involving peripheral blood mononuclear cells (PBMCs) and ovalbumin-sensitized splenocytes indicate that stimulation of TLR7 by AZD8848 inhibits Th2-adaptive responses to allergen via an immune response involving the induction of mediators including interferon alpha (IFN-α) [19-22]. Furthermore, Ikeda *et al*. [23] reported that AZD8848 was effective against allergen-induced airway obstruction and inflammation in guinea-pig models of rhinitis and asthma with weekly as well as acute dosing.

Here, we report the results of two studies. In the first study, increasing single doses of AZD8848 were administered intranasally to healthy subjects and patients with allergic rhinitis. Indices of efficacy and tolerance were monitored. In the second study, AZD8848 was administered intranasally once weekly for five weeks to patients with allergic rhinitis: these individuals were then subjected to repeat allergen exposure and disease activity was monitored focusing on symptoms to establish proof of principle.

## Methods

### Study description

This report comprises two studies evaluating safety and efficacy of single and repeated doses of AZD8848 administered to the nasal airway, both approved by the Regional Ethics Committee and the Swedish Medical Product Agency. They were conducted according to the Declaration of Helsinki and in compliance with Good Clinical Practice, and informed consent was obtained. The studies were of randomized, placebo-controlled, double-blinded, and parallel group designs. Reprotoxicology data was lacking for AZD8848 at the time of these studies and men only were recruited.

### Single ascending dose study (NCT 00688779)

#### Subjects

Eight single ascending intranasal doses of AZD8848 (0.3-600 μg) were given to healthy subjects (n = 48, mean age 26, range 19–44), in groups each involving four individuals receiving AZD8848 and two receiving placebo. Once a maximum tolerated dose was determined, patients with allergic rhinitis (n = 12, mean age 25, range 22–28) received single doses of AZD8848 (30 and 100 μg) and placebo.

Exclusion criteria for healthy individuals were: any relevant disease including seasonal and perennial allergic rhinitis, asthma, clinically relevant structural nasal abnormalities, and upper respiratory tract infection within two weeks prior to the start of the study.

Inclusion criteria for patients were: men with seasonal allergic rhinitis for at least two years, positive skin prick test to birch or grass pollen allergen, asymptomatic condition outside the pollen season, and need for treatment at seasonal allergen exposure.

Exclusion criteria for patients were: any relevant disease including perennial allergic rhinitis, asthma, clinically relevant structural nasal abnormalities, upper respiratory tract infection within two weeks prior to the start of the study, use of topical corticosteroids within four weeks prior to the study and use of antihistamines within one week, and immunotherapy.

#### Study drug

The study product was a solution of AZD8848 (60 mg/g), diluted with sterile buffered saline to concentrations required for each dose level. It was administered using a nasal spray device delivering 50 μL per actuation. The placebo product was isotonic saline. The study product was provided in 10 mL amber glass vials fitted with pump spray devices. In order to assure compliance, study personnel administered the doses. To prevent bronchial airway deposition of the drug, the patients were instructed to exhale against a resistance (30 cm H_2_0), to functionally close the connection between the nasal and bronchial airways, while the study drug was administered.

#### Measurements

Pharmacokinetic parameters were monitored. Furthermore, plasma interleukin-1 receptor antagonist (IL-1Ra) was measured, reflecting type-1 IFN generation downstream from TLR7, and blood lymphocyte counts were carried out. An extensive safety investigation was performed with laboratory measurements (haematology, clinical chemistry, and urine analysis), inspection of the nose (before and 24 hours after dose), vital signs (blood pressure, pulse, and body temperature), and continuous ECG. Finally, AEs were monitored and recorded with information about seriousness, causality, intensity, action taken, recovery, and outcome.

### Repeat challenge/treatment study (NCT 00770003)

#### Subjects

**Figure 1:**
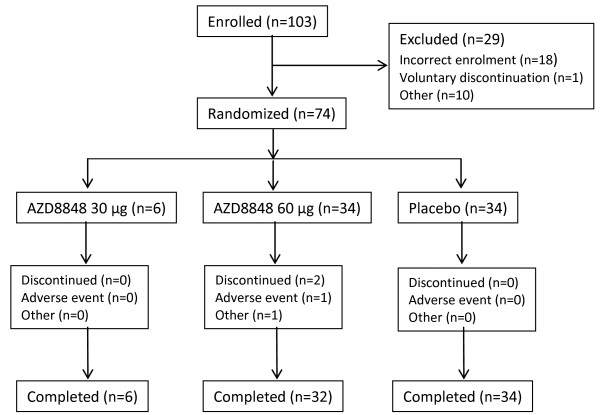
**Patient flow through the repeat challenge/treatment study (participants in Part A as well as Part B).** The progress was characterized by very few dropouts (2.7%).

AZD8848 was given once per week for five weeks. In a first part *(Part A)*, 18 patients were assessed at two dose levels (30 and 60 μg) (mean age 24 years, range 19–39) (Table 1). These individuals were resident at the clinic until 24 hours after each dose and subjected to intense safety monitoring. Of these subjects six received 30 μg AZD8848, six 60 μg AZD8848, and six placebo. In a second part *(Part B)*, 56 patients were examined in an outpatient setting: 28 received 60 μg AZD8848 and 28 received placebo (Table 1). In both groups (Part A as well as B), starting 24 hours after the final dose, daily allergen challenges were performed for seven days. Data from *Part B* were analyzed together with the twelve subjects from *Part A* who either received 60 μg AZD8848 or placebo: progress through the Repeat challenge/treatment study is described in Figure 1. The mean age of these 68 patients was 27 years (range 18–46). Inclusion and exclusion criteria for the patients were the same as described in the dose finding study above for patients with allergic rhinitis.

**Table 1:** Design of the repeat challenge/treatment study (Part A and B)

**Study week**	**1**							**2**							**3**							**4**							**5**							**6**	
	**1**	**2**	**3**	**4**	**5**	**6**	**7**	**1**	**2**	**3**	**4**	**5**	**6**	**7**	**1**	**2**	**3**	**4**	**5**	**6**	**7**	**1**	**2**	**3**	**4**	**5**	**6**	**7**	**1**	**2**	**3**	**4**	**5**	**6**	**7**	**1**	**2**
Treatment	X							X							X							X							X								
Allergen																														X	X	X	X	X	X	X	
Symptoms																														X	X	X	X	X	X	X	X
Plasma	X	X													X	X													X	X							
Nasal lavage	X																												X								X

Nasal symptoms were scored ten minutes after each allergen challenge as well as every morning and evening during the seven days’ allergen challenge series. Note that post challenge and evening symptoms were recorded from day 2 of Study week 5 to day 1 of Study week 6, while morning symptoms were recorded from day 3 of Study week 5 to day 2 of Study week 6. On the first days of Study weeks 1 and 5 nasal lavages were carried out prior to administration of the study drug. The plasma samples were used to monitor safety parameters, pharmacokinetics, and IL-1Ra.

#### Study drug

The study product was AZD8848 (0.3 and 0.6 mg/mL) in buffered saline. It was administered using a nasal spray device delivering 50 μL per actuation. The placebo product had the same composition except for AZD8848. In *Part A*, 30 or 60 μg AZD8848 was administered once per week for five weeks (i.e. 15 or 30 μg per nasal cavity). In *Part B*, 60 μg AZD8848 was given once per week for five weeks (i.e. 30 μg per nasal cavity). In order to assure compliance, study personnel administered all doses. To prevent bronchial airway deposition of the drug, the patients were instructed to exhale against a resistance (30 cm H_2_0), to functionally close the connection between the nasal and bronchial airways, while the study drug was administered.

#### Measurements 1

Pharmacokinetic characteristics were explored only in *Part A* (n = 18). AZD8848 was not expected to be detectable in plasma due to rapid and complete metabolism. Therefore, exposure was monitored through analysis of its acid metabolite. Blood samples were obtained before and 15 minutes and 0.5, 1.0, 1.5, 2.0, and 4.0 hours after the first and fifth study drug administration in *Part A*. Analysis of AZD8848 and its acid metabolite was performed using ethanol extraction followed by liquid chromatography and tandem mass spectrometry.

Plasma samples for analysis of IL-1Ra were obtained during the *Part A* study period prior to and 24 hours following the first, third, and fifth administration of the study drug. In *Part A* (n = 18), the same safety variables were monitored as in the single ascending dose study. In *Part B*, safety monitoring was reduced to vital signs, clinical chemistry, and ECG at pre-dose, prior to the third dose, 24 hours after the fifth dose, and at follow-up. In addition, a flow-cytometric analysis of lymphocyte subsets was performed. AEs were recorded continuously in both groups as described for the single ascending dose study above.

#### Allergen challenge model

In order to establish individually tolerable, repeatable, yet symptom-producing allergen challenge-doses, a nasal titration procedure was performed [24]. In the allergen titration scheme, increasing doses of birch or grass pollen allergen were administrated at ten-minute intervals using a spray-device delivering 100 μL per actuation (Aquagen, ALK-Abelló, Hørsholm, Denmark). One puff was administered into each nostril resulting in effective doses of 100, 300, 1.000, and 3.000 SQ units per nasal cavity. This scheme was followed until the subject responded with at least 5 sneezes or recorded a symptom score of 2 or more on a scale from 0 to 3 for either nasal secretion or nasal blockage. The dose that produced the desired effect was chosen for the allergen challenge series and was given in the morning once daily for seven days, starting 24 hours after the final dose of the study drug.

#### Measurements 2

During the allergen challenge series, the patients scored nasal symptoms every morning and evening. The scores were entered into diary cards and each registration reflected the preceding twelve hours. Nasal secretion and blockage as well as the most severe of sneezing and itching were scored separately on a four-grade scale: 0 = no, 1 = mild, 2 = moderate, and 3 = severe symptoms. The scores were added to a daily total nasal symptom score (TNSS), with separate morning and evening scores. Nasal symptoms were also scored ten minutes post allergen challenge: secretion and blockage were scored as described above, whereas the number of sneezes were counted and transformed into a sneezing score by the investigators: 0 sneezes = 0, 1–4 sneezes = 1, 5–9 sneezes = 2, and 10 or more sneezes = 3. The scores were added to a daily post challenge TNSS.

Nasal saline lavages were obtained at three occasions, i.e. prior to administration of the study drug (baseline observation), 24 hours after the final dose, and 24 hours after the final allergen challenge. Nasal lavages were carried out using a pool-device containing 15 mL fluid and the right nasal cavity was used at all occasions [25]. Each lavage had a five-minute duration. The recovered lavage fluids were centrifuged and the supernatants were homogenized, prepared in aliquots, and frozen (−30°C). The samples were subjected to analysis of α_2_-macroglobulin and tryptase, reflecting plasma exudation and mast cell activity, respectively.

α_2_-Macroglobulin in nasal lavage fluids was measured using a radioimmunoassay sensitive to 7.8 ng/mL. Tryptase was measured using a radioimmunoassay with detection limit of 0.5 ng/mL (Pharmacia-Diagnostics, Uppsala, Sweden). While α_2_-macroglobulin was analysed in the lavage fluids as they were, samples were concentrated ten times before the analysis of tryptase. In plasma, IL-1Ra was measured using a commercial ELISA (Invitrogen, Carlsbad, CA).

### Statistics

In the single ascending dose study, only descriptive statistics were used. Similarly, descriptive statistics only were employed for pharmacokinetic observations and safety variables. In the repeat challenge/treatment study, an ANOVA model was used for comparison between treatments (AZD8848 60 μg vs. placebo): TNSS recorded post challenge and at morning and evening observations were analyzed separately. P-values refer to one-sided hypothesis testing: values <0.05 were considered statistically significant. A multiplicative model was used for biomarkers by log transformation of the response variable and the covariate (i.e. a baseline value). If there was a value below limit of quantification (LOQ) in this analysis, it was estimated to LOQ/2. Treatment differences were estimated from respective model and confidence intervals, and p-values were calculated. In the repeat challenge/treatment study, the six patients receiving AZD8848 30 μg per week were not included in the comparative analysis as they were so few.

## Results

### Single ascending dose study

AZD8848 was not measurable in plasma, whereas its acid metabolite was readily detected. For the 30 and 100 μg doses, respectively, mean maximum plasma concentration (*C*_max_) was 0.27 and 0.65 nmol/L, time to *C*_max_ (*t*_max_) was 15.0 and 22.5 min, and half-life (*t*_1/2_) was 18.4 and 27.6 min in healthy volunteers. Mean *C*_max_ increased with increasing dose levels to a maximum of 1.55 nmol/L with the 600 μg dose. Area under the curve (AUC) increased roughly linearly with ascending dose.

**Figure 2:**
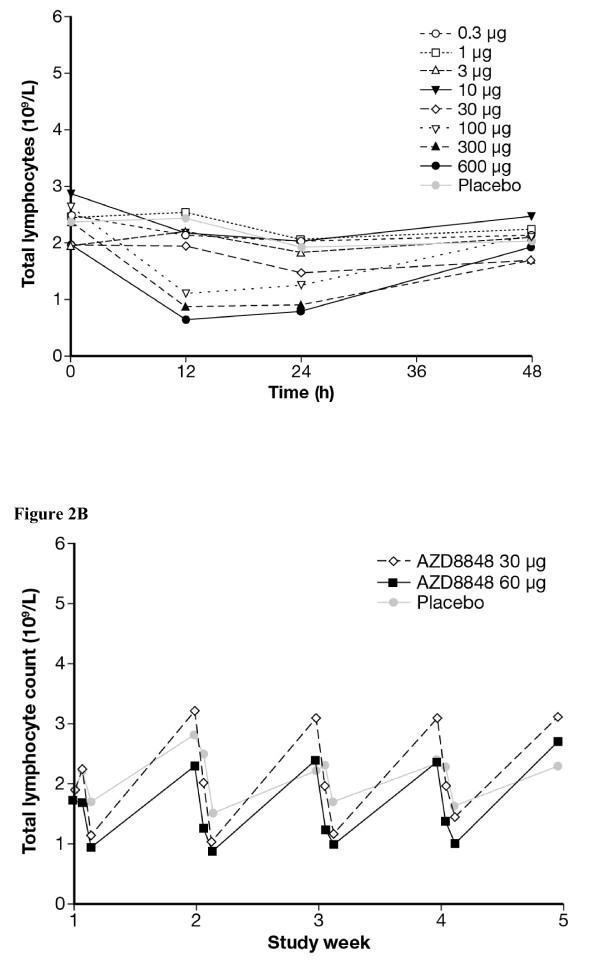
**Mean blood lymphocyte counts in the dose finding study (A) and the repeat challenge/treatment study (B).** AZD8848 produced dose-dependent and repeatable lymphocyte reductions. The reductions were reversible and global, involving CD3+, CD4+, CD8+, and CD20+ cells (data not shown).

The first dose that consistently produced systemic effects in terms of lymphocyte reductions (Figure 2A) and elevations of plasma IL-1Ra (data not shown) was 30 μg. No other specific changes were revealed by the laboratory blood/plasma analysis.

AZD8848 produced dose-dependent influenza-like symptoms starting consistently at 100 μg and leading to termination of dose escalations above 600 μg (Table 2). The second most common effect was mild epistaxis, i.e. blood-admixed nasal secretions.

**Table 2:** Side effects of different doses of AZD8848 in healthy subjects (the single ascending dose study): each group comprised four individuals and the figures indicate the number of subjects reporting a particular experience (by preferred term)

	**Dose (μg)**							
	**0.3**	**1**	**3**	**10**	**30**	**100**	**300**	**600**
Headache	1	0	0	1	0	3	3	4
Epistaxis	1	0	0	0	1	1	1	4
Pharyngeal pain	0	0	0	0	1	0	0	3
Pyrexia	0	0	0	0	0	2	1	3
Rhinorrhea	0	0	0	0	0	2	1	2
Nasal blockage	0	0	0	0	2	1	0	0
Nasal ulcer	0	0	0	0	0	0	2	1
Nasopharyngitis	1	0	0	0	0	0	1	0
Malaise	0	0	0	0	0	0	0	1
Myalgia	0	0	0	0	0	1	1	2

The most common event was flu-like symptoms occurring within 24 hours and disappearing within 48 hours. The second most common event was epistaxis, i.e. blood-admixed nasal secretions. The side effects were dose-dependent and considered causally related to the treatment.

Following the experiments involving healthy individuals, patients with allergic rhinitis (examined out of season) were subjected to administration of AZD8848 (30 and 100 μg). No differences were observed in the response profile to AZD8848 between these patients and the healthy individuals.

### Repeat challenge/treatment study

**Figure 3:**
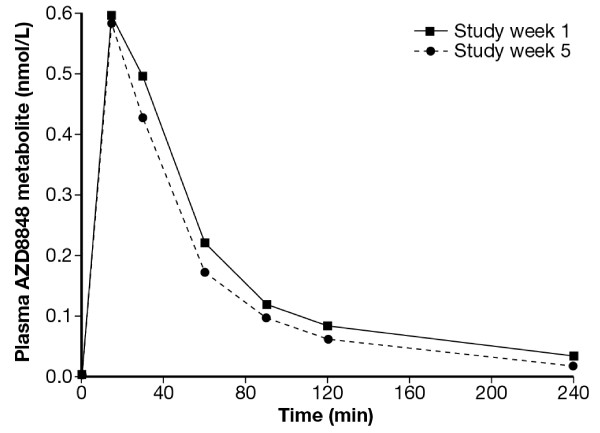
**Mean plasma levels of AZD8848’s acid metabolite from the first group of the repeat challenge/treatment study after administration of 60 μg of the study drug.** The metabolite increased rapidly and returned to baseline levels after four hours.

AZD8848’s acid metabolite was detected at doses of 30 μg (data not shown) as well as 60 μg (Figure 3) For the 60 μg dose, as monitored on Study week 1 and 5, respectively, *C*_max_ was 0.58 and 0.57 nmol/L, *t*_max_ was 17.2 and 20.2 min, and *t*_1/2_ was 0.83 and 1.26 hours.

**Figure 4:**
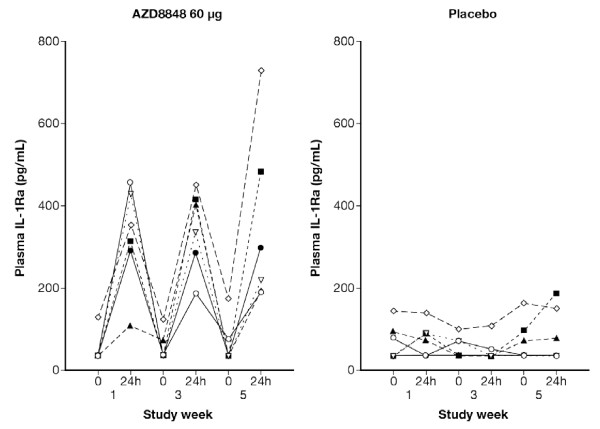
**IL-1Ra in plasma obtained at Study weeks 1, 3, and 5 from the repeat challenge/treatment study (symbols indicate individual values: n = 6).** IL-1Ra increased 24 hours after nasal administration of AZD8848 and the effect was consistent and repeatable (p < 0.001).

Plasma levels of IL-1Ra, reflecting TLR7-induced type-1 IFN production, were consistently increased 24 hours after nasal administration of AZD8848 (Figure 4). For 60 μg, these changes reached statistical significance (p < 0.001). (For 30 μg, comparative statistics was not carried out.)

In agreement with the single dose study, blood lymphocyte counts were consistently reduced after administration of AZD8848 in the repeat challenge/treatment study (Figure 2B). Further analysis indicated that this reflected uniform reductions across different lymphocyte subsets: CD3+, CD4+, CD8+, and CD20+ cells (data not shown).

The safety profile in the repeat dose/treatment study was similar to what is described for the single dose study above, with 35 and 24% reporting flu-like symptoms and blood-admixed nasal secretions, respectively, at treatment with 60 μg AZD8848 (c.f. 6 and 6% for placebo). The symptoms subsided within 24 hours, and did not lead to any individual discontinuations. Two individuals did drop out of the study and both received 60 μg AZD8848: one and two doses, respectively. One case was an AE (anorectal irritation, considered not associated with the treatment) and the other was an individual found to be wrongfully included.

In patients receiving placebo, in agreement with previous observations in this model [24], increases were observed for nasal symptoms, i.e. TNSS: total nasal symptom score, ten minutes after each allergen challenge. Morning and evening total nasal symptoms were also increased and consistent changes were observed from the third study day of the allergen challenge series.

**Figure 5:**
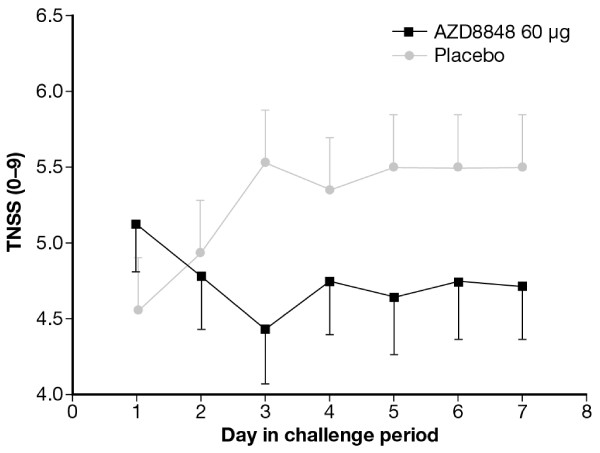
**Mean total nasal symptoms recorded ten minutes after each daily allergen challenge during the seven days’ challenge series.** AZD8848 (60 μg) reduced symptoms recorded ten minutes after challenges from challenge day four and onwards (p < 0.05). Vertical bars indicate SEM.

AZD8848 (60 μg) reduced nasal symptoms recorded ten minutes after allergen challenge from the fourth through the seventh day of the challenge series (i.e. five to eight days after the last dose of AZD8848) with an estimated effect size of 0.74 score units (p < 0.05) (Figure 5). For individual symptoms, reductions were observed for itching (p < 0.05), sneezing (p < 0.05), and blocked nose (p < 0.05), but not for runny nose (data not shown).

**Figure 6:**
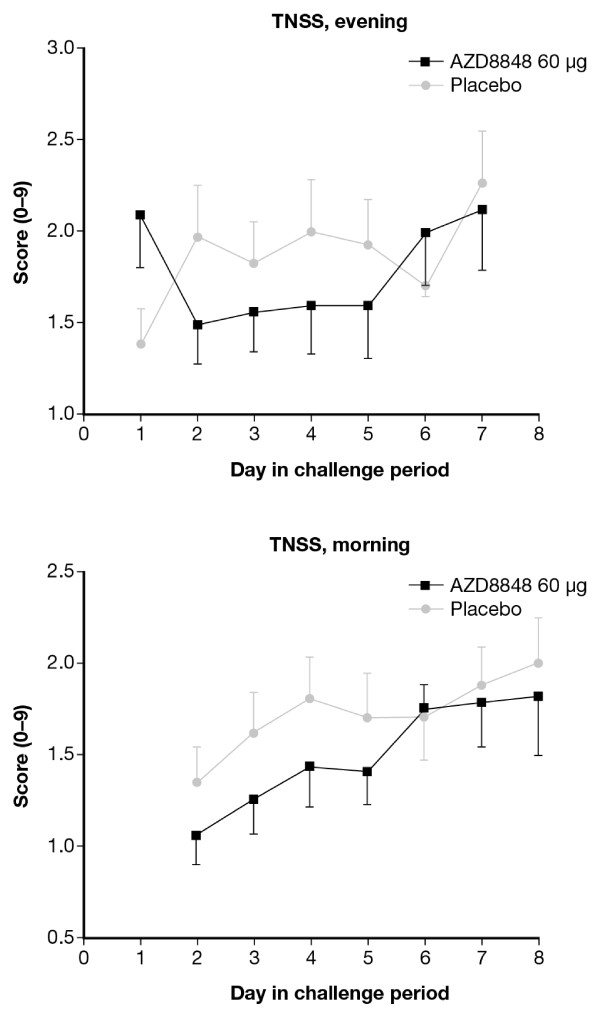
**Mean total nasal morning (upper panel) and evening (lower panel) symptoms, each reflecting the preceding twelve hours, during the allergen challenge series*.*** Symptoms were reduced on challenge days two through five in patients receiving AZD8848 (60 μg) compared with placebo, but these changes failed to reach statistical significance. Vertical bars indicate SEM.

Morning and evening symptoms were numerically reduced on challenge days two through five in patients receiving AZD8848 (60 μg), but these effects failed to reach statistical significance (Figure 6): p > 0.05 for morning as well as evening symptoms.

On the day after the last allergen challenge, levels of α_2_-macroglobulin in nasal lavage fluids, reflecting plasma exudation, were lower in patients who had received 60 μg AZD8848 and this was statistically significant compared to placebo (p < 0.05) (Table 3). Significantly lower levels of mast cell tryptase were also seen in these patients (c.f. placebo) (p < 0.05) (Table 3).

**Table 3:** **Mean ratios (between AZD8848 and placebo) for levels of α**_**2**_**-macroglobulin and tryptase, respectively, in nasal lavages obtained 24 hours after the last allergen challenge in the repeat challenge/treatment study**

**Variable**	**Mean ratio**	**95% CI**	**P-value**
α_2_-Macroglobulin	0.50	0.25-0.97	0.020
Tryptase	0.62	0.35-1.10	0.049

α_2_-Macroglobulin was reduced in patients who had received AZD8848 (p < 0.05, *c.f*. placebo). Similarly, lower levels of tryptase were observed in this group (p < 0.05, *c.f*. placebo).

## Discussion

In these studies we demonstrate that repeated intranasal TLR7 stimulation is associated with reduced responsiveness to allergen in patients with allergic rhinitis. It is the first observation in man where TLR7 has been successfully evaluated as a therapeutic target for allergic airway inflammation.

The TLR7 agonist AZD8848 undergoes very rapid enzymatic degradation by butyrylcholinesterase: *t*_1/2_ in human plasma is estimated to be 20 seconds [19]. In this study, this was reflected by undetectable plasma levels of the substance following nasal administration of doses up to 600 μg, while its acid metabolite, which is 1500 times less potent at the receptor [19], was detectable at doses of 30 μg and above. Nevertheless, flu-like symptoms were reported, which were probably secondary to local TLR7 activation and a subsequent systemic increase in type-1 IFN. This possibility was indicated by the five-fold increase in plasma IL-1Ra following administration of AZD8848, known to reflect type-1 IFN production down-stream to TLR7 (i.e. proof of mechanism) [21,26]. In the context of the hygiene hypothesis, repeated administration of the TLR7 agonist AZD8848 may mimic repeated virus-like stimulation of the immune system.

In the repeat challenge/treatment study, acute symptoms in response to allergen were consistently reduced from the third day of the allergen challenge series in patients receiving the TLR7 agonist when compared to placebo, indicating a sustained effect of at least one week after the final dose of AZD8848. The same pattern was observed for the low-grade morning and evening symptoms, but these changes failed to reach statistical significance. In agreement with a symptom reducing effect, lavage fluid levels of tryptase and α_2_-macroglobulin, reflecting mast cell activity and inflammatory plasma exudation [27,28], were also reduced. Our observations extend recent *in vitro* and animal reports on anti-allergic effects of AZD8848, indicating that repeated TLR7 stimulation reduces the responsiveness to allergen [19-23], and suggest that AZD8848 may be clinically effective in allergic rhinitis.

The observed reduction in responsiveness to allergen might reflect that the immune system was functionally skewed away from a Th2 response. If so, it did probably not represent repolarisation of T-lymphocytes (i.e. a change from Th2 to Th1 phenotype), as this study involved atopic individuals with established populations of memory T-cells with a life span of at least two to three years [29]. Arguably, the outcome was more likely a consequence of a functionally reduced responsiveness of memory Th2 lymphocytes. In this context, for future studies, it would be of interest to examine if prolonged treatment, i.e. a time period sufficient to induce true repolarisation of T-lymphocytes, could produce a more marked anti-allergic effect.

While the repeat challenge/treatment study demonstrated that 60 μg of AZD8848 administered intranasally once weekly for five weeks induced a desired hyporesponsiveness to allergen, likely through activation of TLR7, further studies are warranted to optimize the effect. Preclinical data have indicated that more frequent administration of AZD8848 can produce more marked anti allergic effects (AstraZeneca: data on file). Furthermore, as demonstrated in a Brown Norway rat model of allergic rhinitis/asthma, both nasal and bronchial administration of AZD8848 can reduce the ability of a bronchial allergen challenge to produce bronchial airway eosinophilia and generate IL-13 [19], suggesting the possibility that nasal administration of AZD8848 may be effective in the treatment of asthma.

In this study, safety and tolerability of intranasal AZD8848 was evaluated parallel to the exploration of its anti-allergic effects. None of the patients treated with AZD8848 discontinued the study prematurely due to drug-related AE. Furthermore, standard laboratory indices (haematology, clinical chemistry, and urine analysis) were unaffected by the treatment, except for the anticipated transient reductions in blood lymphocyte counts. Moreover, vital signs (blood pressure, pulse, and body temperature) and continuous ECG were unaffected. However, dose-dependent local side effects were common, albeit of mild intensity. These were dominated by blood-admixed nasal secretions and in these cases nasal inspection revealed superficial mucosal irritations/ulcerations. This effect, and the temporary flu-like symptoms that were experienced by a third of the patients, needs to be further evaluated in order to assess overall tolerability of intranasal AZD8848 as a potential treatment.

The body of knowledge on TLRs is increasing as their distribution and functions are outlined, along with potential associations with specific allergic and airway conditions [30,31] and their treatment, notably specific immunotherapy [32,33]. In the context of established allergic airway conditions, animal observations suggest that stimulation of TLRs (i.e. TLR3, TLR4, TLR7, TLR8, and TLR9) has a general potential to reduce allergen responsiveness. However, focusing on human conditions available observations are scarce. In patients with allergic asthma, a synthetic oligonucleotide containing immunostimulatory CpG motifs (acting on TLR9) was reported to increase the expression of IFN-γ and IFN inducible genes without affecting allergen challenge induced changes [34], possibly reflecting that the dose employed was too low to produce an anti-allergic effect. In a report by Casale *et al*. [35], which focused on tolerability to topical CRX-675 (acting on TLR4), data on efficacy was not given in detail, but a decrease in allergen-induced nasal symptoms was reported in one of four treatment groups compared with placebo. Moreover, it was recently reported that nasal administration of a TLR8 agonist (i.e. VTZ-1463) improved symptoms of allergic rhinitis [36]. Taken together, available information suggests that TLR agonists are valid treatment targets for allergic airway disease.

The natural ligand for TLR7 is single stranded RNA. Accordingly, various respiratory viruses, e.g. influenza, corona, and potentially rhinovirus [37], may activate the receptor. In this context, it is of interest to consider the evidence indicating that respiratory viral infections often heighten the responsiveness to allergen and produce asthma exacerbations [38,39]. The possibility that acute pro-inflammatory effects of TLR7 stimulation may heighten the responsiveness to allergen while later effects may reduce allergen responsiveness suggests that the timing of interventions with TLR7 agonists in relation to allergen exposure is important. Further studies are warranted to explore this and to outline the benefits and risks of treatment with TLR7 agonists in allergic airway disease.

## Conclusion

We conclude that repeated intranasal stimulation of TLR7 by AZD8848 has a potential to affect the immune system in a way that may result in a sustained reduction in the responsiveness to allergen in allergic rhinitis.

## Competing interests

The work described in this manuscript was supported and funded through a collaboration with AstraZeneca and Dainippon Sumitomo Pharma. In the past five years, LG and MA have received project related financial support from AstraZeneca, Schering-Plough, Orexo, HealthCap (Biolipox/Orexo/LTB4 Sweden/CC10 Sweden), Bioglan, and Nares. LG and MA are shareholders in Nares (a company active in the field of allergic rhinitis). In the past five years, AC has received financial support from AstraZeneca and Mediplast. In the past five years, EH has received financial support from AstraZeneca, Grünenthal, and NovoNordisk. GA and AK are employed by AstraZeneca. LE and PN were employed by AstraZeneca when the study was conducted. CAE, JD, ILS, and HW declare no competing interests.

## Authors’ contributions

Conception, design and management: LG, GA, AC, LE, EH, PN. Patient examination: LG, CAE, MA, AC, JD, EH, PN, ILS, HW. Data input, drafting, revision and/or approval of manuscript: All. Statistics: LG, AC, AK. All authors read and approved the manuscript.
